# NbSe_2_ Nanosheets/Nanorolls Obtained via Fast and Direct Aqueous Electrochemical Exfoliation for High-Capacity Lithium Storage

**DOI:** 10.1021/acsaem.3c00893

**Published:** 2023-06-16

**Authors:** Daniel F. Carrasco, Sergio García-Dalí, Silvia Villar-Rodil, José M. Munuera, Encarnación Raymundo-Piñero, Juan I. Paredes

**Affiliations:** † Instituto de Ciencia y Tecnología del Carbono, INCAR-CSIC, Francisco Pintado Fe 26, Oviedo 33011, Spain; ‡ CNRS, CEMHTI UPR3079, Univ. Orléans, 1D Avenue de la Recherche Scientifique, Orléans 45071, France

**Keywords:** layered transition-metal dichalcogenides (LTMDs), NbSe_2_, VSe_2_, electrochemical exfoliation, nanorolls, energy storage

## Abstract

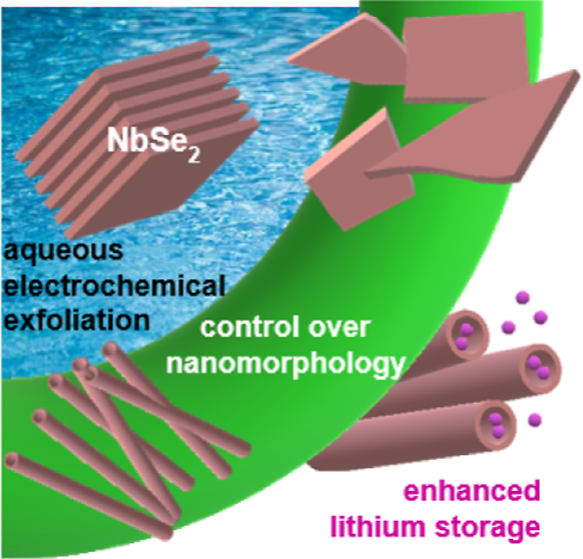

Layered transition-metal dichalcogenides (LTMDs) in two-dimensional (2D) form are attractive for electrochemical energy storage, but research efforts in this realm have so far largely focused on the best-known members of such a family of materials, mainly MoS_2_, MoSe_2_, and WS_2_. To exploit the potential of further, currently less-studied 2D LTMDs, targeted methods for their production, preferably by cost-effective and sustainable means, as well as control over their nanomorphology, are highly desirable. Here, we report a quick and straightforward route for the preparation of 2D NbSe_2_ and other metallic 2D LTMDs that relies on delaminating their bulk parent solid under aqueous cathodic conditions. Unlike typical electrochemical exfoliation methods for 2D materials, which generally require an additional processing step (e.g., sonication) to complete delamination, the present electrolytic strategy yielded directly exfoliated nano-objects in a very short time (1–2 min) and with significant yields (∼16 wt %). Moreover, the dominant morphology of the exfoliated 2D NbSe_2_ products could be tuned between rolled-up nanosheets (nanorolls) and unfolded nanosheets, depending on the solvent where the nano-objects were dispersed (water or isopropanol). This rather unusual delamination behavior of NbSe_2_ was explored and concluded to occur via a redox mechanism that involves some degree of hydrolytic oxidation of the material triggered by the cathodic treatment. The delamination strategy could be extended to other metallic LTMDs, such as NbS_2_ and VSe_2_. When tested toward electrochemical lithium storage, electrodes based on the exfoliated NbSe_2_ products delivered very high capacity values, up to 750–800 mA h g^–1^ at 0.5 A g^–1^, where the positive effect of the nanoroll morphology, associated to increased accessibility of the lithium storage sites, was made apparent. Overall, these results are expected to expand the availability of fit-for-purpose 2D LTMDs by resorting to simple and expeditious production strategies of low environmental impact.

## 1 Introduction

The emergence of graphene in 2004 signaled the beginning of the now vast field of two-dimensional (2D) materials, the breadth of which pertains to the large number of different known 2D systems, their possible combinations (2D heterostructures), and the exotic physical phenomena they can give rise to, as well as to the variety of technological domains where they can be potentially applied.^1,2^ One such relevant domain is that of electrochemical energy storage (EES), where many 2D materials (typically, those having sufficient electrical conductivity) can be used as efficient electrodes for various types of supercapacitors^2^ and batteries,^3^ including metal-ion, metal-sulfur, and metal-air batteries. In addition to the distinct advantages that a given 2D system might exhibit on account of its particular chemical composition and structural configuration, 2D materials possess some overarching features that make them especially attractive for EES applications, most notably, high specific surface areas and extremely thin (usually <10 nm) sheet-like morphologies.^3^ Such features are expected to be conducive to promoting charge storage by facilitating extensive interactions with, e.g., ions and redox-active species, as well as by expediting mass transport through the electrode, particularly when the thin sheets are of limited lateral dimensions (e.g., below a few micrometers).

Over the last decade, intensive research efforts have been made to unlock the full potential and advance the implementation prospects of 2D materials in EES devices.^2−4^ However, these efforts have preferentially, although not exclusively, focused on a small subset of 2D materials, which mainly includes graphene and its derivatives,^5^ a few layered transition-metal dichalcogenides (LTMDs), MXenes, and Xenes (mostly MoS_2_, Ti_3_C_2_T_*x*_, and phosphorene, respectively),^6−8^ as well as some 2D metal oxides and hydroxides.^9^ Not surprisingly, many of them are readily accessible materials, with techniques for their production and processing in significant amounts being already relatively mature. Thus, to uncover the possibilities and exploit the promise of other, currently less-explored 2D materials for EES, suitable targeted preparation methods should be developed in parallel.^4^ Another relevant issue in the use of 2D materials as electrodes for EES concerns their actual nanomorphology and aggregation state in the electrode. 2D materials are often obtained in the form of stand-alone, largely unfolded nanosheets, e.g., in colloidal dispersion, but their processing into electrodes can easily lead to widespread nanosheet restacking and, consequently, to a dramatic decrease in the available surface area and to a deterioration of the ion diffusion kinetics.^10^ This issue can be alleviated by, among other strategies, assembling the nanosheets into suitable nanomorphologies, such as loose aggregates (e.g., nanoflowers), hollow/porous structures (nanorolls, nanospheres, porous gels, etc.), or highly crumpled films.^11^ Therefore, straightforward approaches that allow access to these types of nanomorphologies with 2D materials are also highly desirable.

As a member of the 2D LTMD family, NbSe_2_ nanosheets are a very attractive material for EES applications. Different to the case of the most commonly studied LTMDs, such as MoS_2_, WS_2_, and MoSe_2_, which are electrically semiconducting compounds in their thermodynamically stable phase (2H phase), NbSe_2_ exhibits a metallic nature that should favor its use in such applications.^12^ Indeed, although still rather limited in number, some reports have in recent years disclosed the potential of NbSe_2_ nanosheets/nanostructures, either alone or in combination with other materials, as a suitable electrode for Li-ion,^13−15^ Na-/K-ion,^16−18^ and multivalent-ion^13,19^ storage in batteries, as well as for Li–S batteries^20^ and supercapacitors.^21,22^ However, also in contrast with the most widespread LTMDs, the pool of available methods for producing NbSe_2_ nanosheets in significant amounts has so far remained quite small, which in turn has limited the range of practically available 2D NbSe_2_ materials. For example, top–down methods essentially boil down to the well-known, generic exfoliation of the bulk material in proper organic solvents driven by ultrasound^23^ and to electrochemical exfoliation with electrolytes based on lithium or alkylammonium salts, also in organic solvents.^24−26^ Likewise, control over the nanomorphology of 2D NbSe_2_ has been mainly restricted to nanoflower-like structures obtained by solvothermal synthesis.^14^ Hence, strategies that allow access to NbSe_2_ nanosheets as well as some control of their nanomorphology without resorting to the use of organic media (e.g., more sustainable, water-based strategies) would be an important asset to further the prospects of this material in EES and other applications.

Here, we report a straightforward approach for the production of NbSe_2_ nanosheets via an electrochemical delamination route carried out in an aqueous electrolyte. We also show that, depending on the specific solvent where the exfoliated product is dispersed (water or isopropanol), the obtained nanosheets can be preferentially rolled-up into 1D nano-objects (nanorolls) or kept in an unfolded state. A mechanism based on redox processes is proposed to account for the somewhat unusual, smooth electrolytic delamination of the starting bulk NbSe_2_ material. Such a mechanism applies to other metallic LTMDs, allowing their straightforward delamination, as shown here for NbS_2_ and VSe_2_. Likewise, the 2D NbSe_2_ nanorolls and unfolded nanosheets are both investigated as an electrode material for Li-ion storage, thus affording a direct comparison of the effect of nanomorphology on the EES performance. In particular, the nanorolls are seen to reach quite high Li-ion storage capacity values, making them a competitive anode material for Li-ion batteries. Thus, by introducing a simple, water-based method for producing 2D NbSe_2_ with controlled nanomorphology, the present results should expand the scope of less-studied LTMDs in EES and beyond.

## 2 Results and Discussion

### 2.1 General Aspects of the Cathodic Delamination of NbSe_2_ in Aqueous Medium

**Figure 1:**
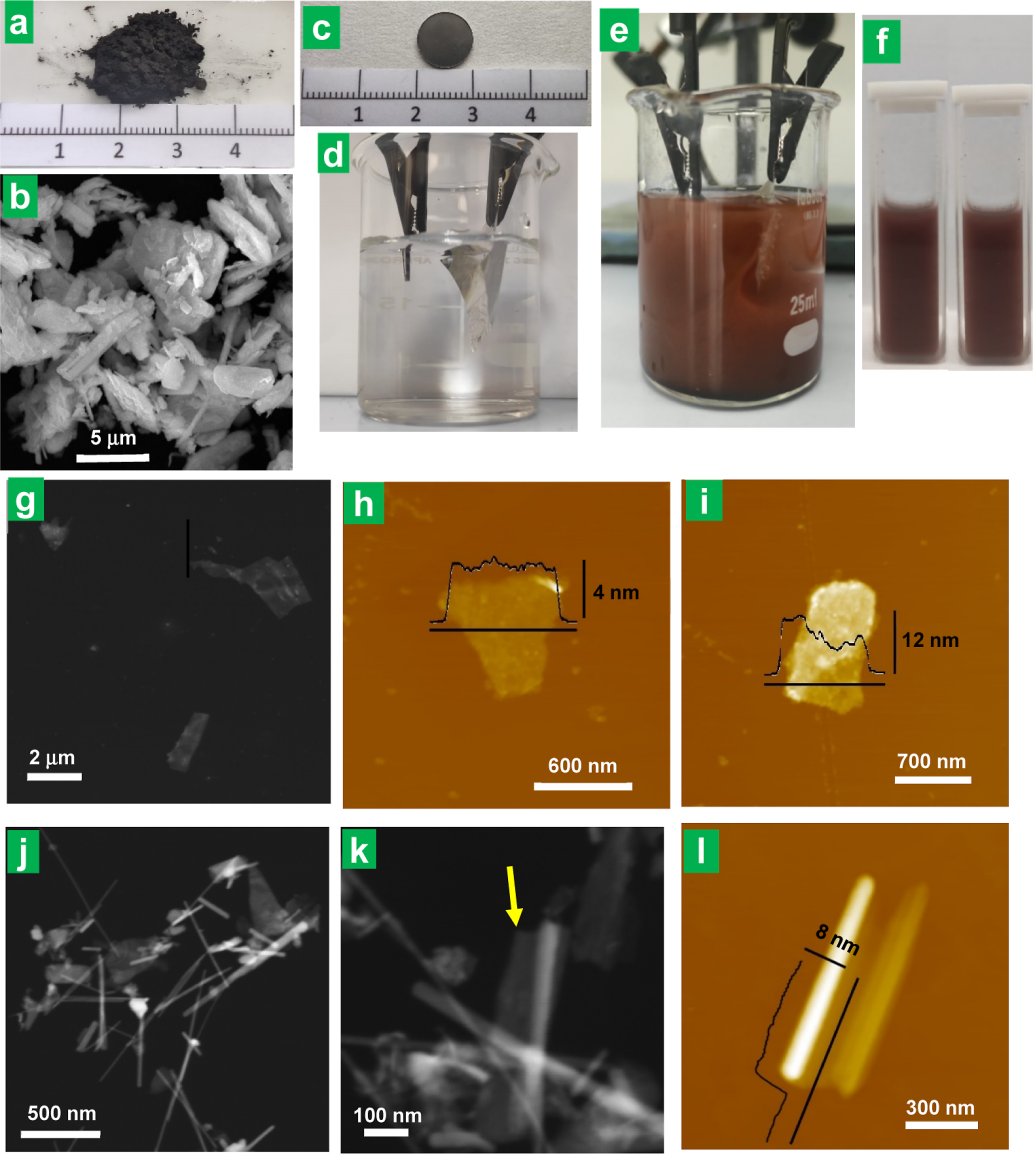
(a) Digital photograph and (b) FE-SEM micrograph of commercial NbSe_2_ powder. Digital photographs of (c) the NbSe_2_-graphite foil cathode and the experimental setup for electrolytic exfoliation (d) before and (e) after application of a negative voltage (−10 V) to the NbSe_2_-graphite foil cathode. (f) Digital photographs of the cathodic exfoliation product colloidally dispersed in isopropanol (left) and water (right). Typical STEM (g) and AFM images (h,i) and of the cathodically derived product deposited from its dispersion in isopropanol. (j,k) STEM images of the cathodic product deposited from its dispersion in water. The arrow in *k* points to a site where the sheet is incompletely rolled-up. (l) AFM image of a partially unfolded nanoroll.

An electrochemical delamination route was implemented for the preparation of NbSe_2_ nanosheets from their corresponding bulk material in powder form [details of the procedure are given in the Experimental Section (see Supporting Information)]. In an optimized procedure, a certain amount of commercial NbSe_2_ powder (Figure 1a), made up of micrometer- and submicrometer-sized particles [see the field-emission scanning electron microscopy (FE-SEM) image in Figure 1b], was compacted onto a circular piece of graphite foil by means of a hydraulic press (Figure 1c). The graphite foil served as an electrically conductive supporting substrate for the LTMD and acted as the cathode in an electrolytic setup that made use of platinum foil as the anode. Both electrodes were immersed in an aqueous 0.3 M KNO_3_ solution in a parallel configuration and kept at a distance of ∼2 cm from each other (Figure 1d). The side of the graphite foil coated with NbSe_2_ was directly facing the platinum counter electrode. Upon application of a negative voltage (−10 V) to the NbSe_2_-graphite foil electrode for a few minutes, lustrous gray particles were seen to detach from it and to quickly sediment at the bottom of the electrolytic cell. At the same time, a reddish-brown substance was also released from the cathode surface, but this remained suspended in the electrolyte instead (Figure 1e; see also Movie S1 in the Supporting Information). Such a substance, as well as the gray particles, necessarily originated from the NbSe_2_ component of the cathode because they were not observed when the naked graphite foil was negatively biased. The latter only underwent a slight swelling and released just a few black particles that floated atop the electrolytic solution (see Movie S2 in the Supporting Information). Indeed, aqueous cathodic delamination of graphite in significant extent is known to require treatments on the scale of hours, rather than minutes, and the use of more complex, specific electrolytes.^27^ Moreover, both the reddish-brown substance and the gray particles were also generated when stand-alone, pressed NbSe_2_ pellets were used as the cathode, i.e., in the absence of the graphite foil support (see Movie S3 in the Supporting Information). However, such stand-alone pellets were relatively brittle and thus more difficult to handle than their graphite foil-supported counterparts. After completion of the cathodic treatment in just a few minutes, the reddish-brown substance in the electrolyte was collected (the gray sediment discarded), washed with deionized water [three cycles of (i) sedimentation via centrifugation or overnight resting and (ii) resuspension in water], dried under vacuum at room temperature, and then stored for further use. This product could be colloidally dispersed in isopropanol and water (Figure 1f) with the aid of a vortex mixer or a brief (1 or 2 min) sonication step, although its stability was generally rather limited, with most of the material sedimenting in several hours. Nonetheless, the sediment could be easily resuspended for an indefinite number of times, indicating that an irreversible agglomeration of the colloidally dispersed product was not taking place.

Figure 1g shows a typical scanning transmission electron microscopy (STEM) image of the cathodically derived product from its dispersion in isopropanol, where it can be noticed that the material was made up of submicrometer-sized nanosheets of irregular polygonal profile, showing lengths mostly in the ∼300–600 nm range. As determined by atomic force microscopy (AFM; Figure 1h,i), the nanosheets were typically between 4 and 12 nm in thickness. Based on the above observations, it is reasonable to conclude that these 2D objects were obtained from the delamination of bulk NbSe_2_ during the cathodic treatment. If we assume 0.628 nm to be the thickness of a NbSe_2_ monolayer,^28^ then the present nanosheets would be nominally several (∼6–19) monolayers thick, according to the above AFM results (Figure 1h,i). Nonetheless, this result will need to be refined in the light of further data, as discussed below. However, the actual morphology of the delaminated nano-objects could also be modulated by a proper selection of the dispersing solvent. Figure 1j presents a STEM image of the cathodic product deposited from its dispersion in water, instead of isopropanol. In this case, while nanosheets were still spotted in significant numbers, the sample was largely dominated by 1D entities that were about 500–700 nm long and ∼20 nm wide. Closer inspection of the 1D objects (Figure 1k) revealed them to possess a tubular morphology and suggested that they were formed by the rolling-up of nanosheets to give nanorolls (note the incompletely rolled-up sheet at the site marked by an arrow in Figure 1k). Further evidence of this point is given in the AFM image of Figure 1l, where a partially unrolled nanoroll can be noticed. The two ends of the corresponding nanosheet appeared to be rolled up, with only its middle section unrolled. From the unrolled section, the thickness of the nanosheet was estimated to be ∼7–8 nm. It is worth noting that this nanosheet was initially fully rolled up (a single 1D object was seen during the first AFM scan, image not shown), but a partial unrolling apparently took place at some point of the measurement, likely as a result of perturbations induced by interaction with the AFM tip.

The transformation between unfolded nanosheet and nanoroll morphology of the present cathodically delaminated materials appeared to be reversible through solvent exchange. For example, the as-obtained, dried cathodic product first dispersed in isopropanol yielded unfolded nanosheets, but if isopropanol was exchanged by water (via several cycles of sedimentation by centrifugation and resuspension in water), nanorolls became the dominant nanomorphology, and vice versa (see Figure S1 in the Supporting Information). Not surprisingly, nanorolls were prevalent in the as-obtained, dried cathodic product that was not subsequently dispersed in any solvent (Figure S2), given that this product was originally processed in water. It is also worth noting that such a solvent-driven control of the nanomorphology was not exclusive of the cathodically derived material. Indeed, direct sonication of bulk NbSe_2_ powder in isopropanol and water also afforded suspensions made up of unfolded nanosheets and rolled-up nanotubes, respectively (Figure S3). However, the cathodic delamination strategy turned out to be a much more efficient process than direct sonication. For instance, a delamination yield of ∼16 wt % could be obtained with the cathodic process in just a few minutes (at the expense of just ∼0.7 A h/g), whereas direct sonication only gave a yield of ∼6 wt % after 6 h. Generally speaking, it is not possible to establish a comparison in terms of delamination yield with previous examples of NbSe_2_ electrochemical exfoliation in the literature as this data is not usually reported.^24,26^ In some instances, the efficiency of the exfoliation is discussed in terms of exfoliation degree, i.e., the percentage of monolayers obtained over the whole exfoliated material^26^ but, of course, this does not allow to know which wt % of the starting material has been exfoliated. We note that the delamination yield of ∼16 wt % found here could be further increased if, instead of discarding the gray sediment from the electrolytic exfoliation of NbSe_2_ (which is none other but nonexfoliated NbSe_2_), it was recovered and pressed again onto a graphite foil disc to be reused as an anode in successive exfoliation cycles.

The effect of different electrolytic parameters on the amount of delaminated product was also investigated. Taking the 0.3 M KNO_3_ electrolyte and a bias voltage of −10 V as the benchmark treatment conditions, we observed that both lower (e.g., 0.1 M) and higher (e.g., 1 M) electrolyte concentrations clearly led to smaller amounts of delaminated products. In the former case, delamination was relatively slow and generated little reddish-brown substance in the aqueous solution. In the latter, a much more vigorous process was noticed, but this only resulted in a very quick detachment of gray particles that sedimented at the bottom of the solution, with no or very little reddish-brown substance being released. Under such conditions, the detaching particles probably loose their electrical contact to the cathode so quickly that there is virtually no time for them to delaminate to any significant extent. The magnitude of the bias voltage had a similar effect, so that both smaller (e.g., −5 V) and larger (e.g., −20 V) voltages gave rise to lower delamination efficiencies. The amount of the delaminated product also decreased when changing the cation in the electrolyte from K^+^ to Na^+^ or Li^+^, which was ascribed to the reduced ability of the latter ions in hydrated form to reach and intercalate the LTMD due to their lower ionic conductivity and larger size.^27^ Changing the electrolyte anion to, e.g., Cl^–^ had no apparent effect on the delamination efficiency. However, the use of anions that are highly oxidizing or can readily generate highly oxidizing species in the electrolyte should be avoided as these could then attack the oxidation-prone NbSe_2_ (see below). In this regard, the Cl^–^ anion can be expected to anodically oxidize during the electrolytic treatment to give Cl_2_ and then hypochlorous acid (HOCl) upon reaction with water, which is a rather oxidizing species. By contrast, the reduction potential of the NO_3_^–^ anion is lower than that of HOCl (0.96 vs 1.48 V, relative to the standard hydrogen electrode),^29^ making it a weaker oxidizing species and thus a better choice as the electrolyte anion for NbSe_2_ exfoliation.

### 2.2 Physicochemical Characterization of Cathodically Delaminated NbSe_2_

**Figure 2:**
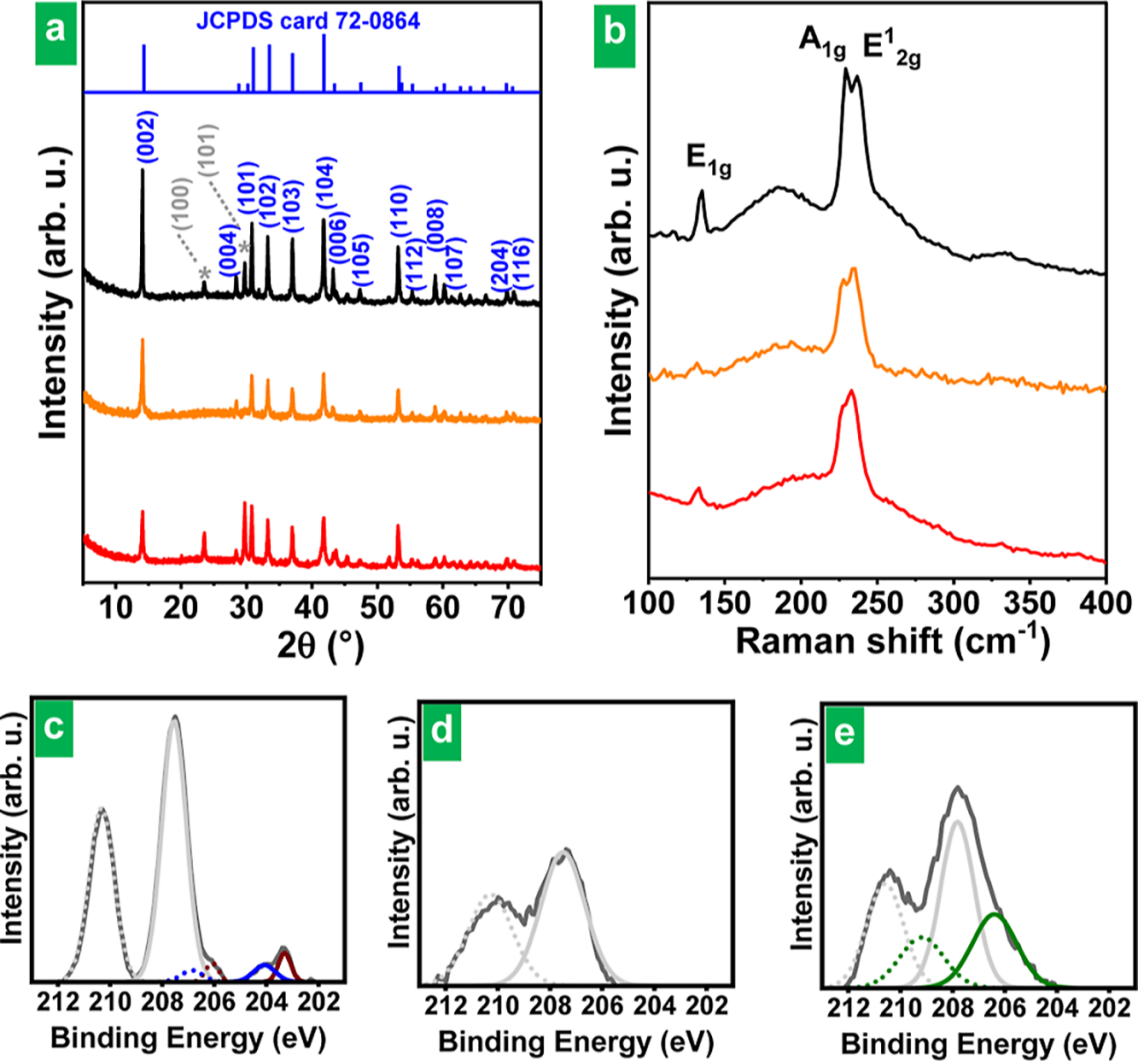
XRD pattern (a) and Raman spectrum (b) of the starting bulk NbSe_2_ powder (black trace) and the cathodically delaminated product processed in water only (orange trace) and in isopropanol (red trace). 2H-NbSe_2_ XRD peaks have been labeled according to JCPDS card 72-0864 (blue trace and labels), while the peaks labeled in gray and marked with an asterisk correspond to crystalline selenium in trigonal phase, namely, to 2θ values of 23.5 and 29.7° corresponding to the crystal planes (100) and (101), respectively. Background-subtracted high-resolution Nb 3d XPS spectrum of (c) the starting bulk NbSe_2_ powder and the cathodically delaminated product processed in (d) water only and (e) in isopropanol. Niobium appears in different oxidation states and/or chemical environments: Nb(V) from orthoniobates or polyoxoniobates (gray trace); Nb(IV) from NbSe_2_ (wine trace) and in NbO_2_ (green trace); and Nb(II) in NbO (blue trace). The Nb 3d_3/2_ and Nb 3d_5/2_ components are graphed with solid and dotted lines, respectively.

The identity of the delaminated products was assessed by X-ray diffraction (XRD), Raman spectroscopy, and X-ray photoelectron spectroscopy (XPS). Possessing different probing depths, each of these characterization techniques gathers partial information on the material, which must be combined to generate a global picture (see Figure S4). The XRD pattern of the starting bulk NbSe_2_ powder (Figure 2a, black trace) exhibited an array of well-defined, sharp diffraction peaks that was consistent with the material being 2H-phase NbSe_2_ (hexagonal crystal structure with space group *P*6_3_/*mmc*).^30^ A couple of additional peaks, marked with an asterisk in the diffractogram, indicated the presence of elemental selenium of trigonal phase (t-Se) as an impurity in the material.^31,32^ For NbSe_2_, the strong (002) reflection located at ∼14.1° (2θ) indicated the interlayer distance in the LTMD to be of 0.628 nm. Most, if not all, of the diffraction peaks characteristic of 2H-phase NbSe_2_ were also present in the cathodically delaminated product, whether the latter was predominantly in the form of nanorolls (i.e., processed in water only; Figure 2a, orange trace) or of unfolded nanosheets (upon processing in isopropanol; Figure 2a, red trace). Some t-Se was also noticed in the XRD patterns of some of the delaminated products. As will be explained below, although elemental Se is indeed present in the exfoliated products, it is not necessarily in crystalline t-Se form. It can be in amorphous, S_8_ form, which is not detectable by XRD. Although the intensity of the (002) peak could be maybe expected to decrease relative to that of the other peaks upon exfoliation^30^ (and finally disappear in the complete exfoliation, down to monolayer), the simultaneous decrease in the lateral size of the NbSe_2_ entities will also diminish the intensity of the XRD peaks corresponding to other spatial directions, and thus the final effect in the relative intensities is uncertain.

Figure 2b (black trace) shows a typical Raman spectrum of bulk NbSe_2_ powder in the 100–400 cm^–1^ wavenumber range, where the characteristic bands (signature peaks) for the 2H phase of this LTMD are known to be located.^33,34^ These include the intense and sharp A_1g_ (∼230 cm^–1^) and E^1^_2g_ (∼240 cm^–1^) peaks, which correspond, respectively, to an out-of-plane phonon of the chalcogen atoms and an in-plane phonon of both the metal and chalcogen atoms. The weaker E_1g_ peak (∼135 cm^–1^) is an in-plane phonon of the chalcogen atoms that is thought to be activated only at edge planes. In addition, a broad band that could be assigned to the so-called soft mode was noticed at about 180 cm^–1^. The latter is a second-order feature involving two-phonon scattering processes, and its relatively high intensity is connected to the fact that NbSe_2_ exhibits charge density wave states at low temperatures.^35,36^ These Raman bands have been shown to be also present, with very minor variations (e.g., a shift in peak positions of just a few inverse centimeters, if any), in atomically thin NbSe_2_.^37,38^ In fact, such bands were clearly noticed in the cathodically delaminated product obtained here, whether it was processed in water only or transferred to isopropanol (Figure 2b, orange and red traces, respectively). Nonetheless, the Raman features of this product were largely but not totally coincident with those expected for (bulk or atomically thin) NbSe_2_. Specifically, in the delaminated material, the peak at ∼240 cm^–1^ (i.e., the E^1^_2g_ band frequency of 2H-NbSe_2_) was somewhat more intense than its 230 cm^–1^ counterpart (A_1g_ band frequency of 2H-NbSe_2_), whereas this is not usually the case with the E^1^_2g_ and A_1g_ bands for pure NbSe_2_ (e.g., see the spectrum of bulk NbSe_2_ in Figure 2b).^33,35,37,38^ We interpret such distinct Raman feature of cathodically delaminated NbSe_2_, relative to those expected for the pure LTMD, to arise from a partial oxidation of the obtained nanosheets/nanorolls. The surface of bulk NbSe_2_ is known to slowly oxidize under ambient conditions, giving Nb(V) species, which are mainly thought to be amorphous Nb_2_O_5_ and elemental Se.^39,40^ This reaction can be accelerated with external stimuli, such as heat or light.^41,42^ Surface oxidation readily develops on 2D NbSe_2_ nanosheets as well.^22,43^ Moreover, as will be shown below, the present cathodic treatment of NbSe_2_ leads to surface oxidation of the delaminated objects. However, the unusual Raman features of the delaminated products cannot be ascribed to the presence of Nb_2_O_5_, either in crystalline or in amorphous form (see Figure S5). In fact, Nb_2_O_5_ exhibits other intense features outside the 100–400 cm^–1^ range, particularly strong bands at around 600–700 cm^–1^,^44^ which we detected in commercial samples of both crystalline and amorphous Nb_2_O_5_ but totally absent in the cathodically delaminated NbSe_2_ material (see Figure S5). Instead, the unusual Raman features of the delaminated products most likely arise from the presence of elemental Se as the other product of NbSe_2_ oxidation. In fact, the Raman spectrum of t-Se, which was detected here by XRD (Figure 2a), is known to be dominated by a band at 235–240 cm^–1^ (A_1_ phonon), whereas Se_8_ rings in amorphous Se (a-Se) give rise to a peak at around 260 cm^–1^ (intraring stretching vibration).^45,46^ Elemental Se, both in crystalline and amorphous form, was therefore very likely responsible for the slightly altered Raman features of cathodically delaminated NbSe_2_ (a Raman spectrum of elemental selenium will be shown below regarding the discussion of the exfoliation mechanism).

Some (native) surface oxidation was also expected on the starting bulk NbSe_2_ particles, but the thickness of the corresponding layer of oxidation products was probably small compared to the probing depth of the Raman technique (a few tens of nanometers for NbSe_2_^33^, see Figure S4). For this reason, the intensity of the Raman features resulting from such products (i.e., those of elemental Se) in bulk NbSe_2_ was negligible relative to those coming from the LTMD proper (Figure 2b, black trace). In the delaminated materials, the amount of surface oxidation products probed relative to that of (nonoxidized) NbSe_2_ should be considerably larger due to the nanometric thickness of the nanosheets/nanorolls (see Figure S4). Hence, their Raman bands should be easier to detect (Figure 2b, orange and red plots). In agreement with previous work,^47^ further oxidation of the delaminated nanosheets/nanorolls could be triggered by repeatedly measuring a given area with the Raman laser beam. This effect was clearly noticed from the progressive rise of the 240 and 260 cm^–1^ peaks when spectra were consecutively recorded on the same spot (not shown). In consequence, to avoid altering the exfoliated samples throughout the measurements, the spectra were obtained with low laser powers (below 0.5 mW) and short measurement times (a few tens of seconds).

The presence of a surface layer of oxidation products both on the starting bulk NbSe_2_ powder and on its cathodically delaminated counterpart was disclosed by a much more surface-sensitive technique (smaller probing depth), namely, XPS (see Figure S4). The high-resolution core-level Nb 3d spectra of the bulk powder as well as that of the exfoliated nanorolls and nanosheets are presented in Figure 2c–e, respectively. In all cases, the spectral envelopes were dominated by components located at ∼207.5 and ∼210.2 eV (gray traces in Figure 2c–e), which can be assigned to the 3d_5/2_ and 3d_3/2_ levels of Nb(V), respectively, and were thus indicative of Nb oxide species on the surface of the samples.^19^ Much weaker features consistent with 2H-phase NbSe_2_, i.e., the components located at about 203.2 [Nb(IV) 3d_5/2_] and 206.0 [Nb(IV) 3d_3/2_] eV,^19^ were observed in the spectrum of the bulk powder (wine traces in Figure 2c). Niobium oxides with oxidation states lower than V were also detected (blue and green traces in Figure 2c,e, respectively). This result agrees with previous reports in the literature on XPS characterization of NbSe_2_ nanosheets where Nb(V) is either the only^14^ or the majoritarian component detected^24^ in the Nb 3d spectrum. The core-level O 1s spectra of these materials were consistent with most of the surface Nb being in the form of oxide, e.g., orthoniobates, polyoxoniobates, or Nb_2_O_5_ (Figure S6). In agreement with the detection of elemental Se by XRD and Raman spectroscopy (Figure 2a,b), XPS also revealed the presence of this species in the bulk and delaminated materials (see Se 2p core-level spectra in Figure S7).

In the case of the delaminated nanosheets/nanorolls, we assume that the surface layer of oxidation products mostly formed during their preparation by the cathodic treatment (see below). Moreover, it can be inferred that just one or two of the outermost monolayers in the NbSe_2_ nanosheets (as well as their rolled-up counterparts) eventually got oxidized to give Nb oxide and elemental Se species on the surface. These oxidized layers would sandwich an inner section containing a few or several nonoxidized NbSe_2_ monolayers. Such a conclusion was mainly supported by two facts. First, the thickness of the delaminated nanosheets was in the 4–12 nm range. Second, upon oxidation, the thickness of NbSe_2_ slabs/nanosheets has been shown to increase substantially, up to a factor of ∼3.^41,47^ Hence, considering that a pristine NbSe_2_ monolayer is ∼0.6 nm thick,^28^ the layer of oxidation products in the delaminated nanosheets should not have originated from, e.g., the three outermost NbSe_2_ monolayers, as these would have led to nanosheets considerably thicker than those actually measured here. Rather, the nanosheet thickness would be consistent with such an oxidation layer coming from one or two NbSe_2_ monolayers. Moreover, the thickness of this oxidation layer (i.e., a few nanometers) would be similar to values given in the literature for the native oxide in 2D NbSe_2_ and NbS_2_ nanostructures,^48,49^ as well as to the typical probing depth of the XPS technique,^50^ implying that most, if not all, of the XPS signal should originate from the oxidation layer (see Figure S4), as demonstrated in Figure 2c–e and reported in previous works on NbSe_2_ nanosheets.^14,24^ However, as explained above, the deeper probe depth of a few tens of nanometers of Raman spectroscopy will allow detecting both the oxide layer and the 2H-NbSe_2_ core of a number of thin nanosheets (4–12 nm thick, see Figure 1h,i) constituting the probed films (see Figure S4), while less proportion of the oxide layer (presumably only one oxidized surface) will contribute to the Raman signal for thicker, unexfoliated starting NbSe_2_ powder (Figure 2b). The larger probe depth of XRD will allow detecting the surface oxide layer and the unmodified 2H-NbSe_2_ core for both the exfoliated objects and the starting powders (Figures 2a and S4). We note that the appearance of an oxidation layer is not avoided by using lower voltages for the electrochemical delamination as the layer is present even in the case of the starting, nonelectrochemically treated material. In fact, in previous reports in the literature for the case of electrochemical exfoliation of NbSe_2_ in organic media, even when the electrochemical parameters were adjusted to minimize structure degradation, the main Nb species detected by XPS was still Nb(V).^24^

### 2.3 Rationalizing the Aqueous Cathodic Delamination of NbSe_2_ and the Morphology of the Delaminated Products

The above results demonstrate that bulk NbSe_2_ can be readily exfoliated by electrochemical means based on a simple and expeditious aqueous process to give nanometer-thick sheets that can be switched between unfolded and rolled-up morphologies. We note that, while the protocols reported in the literature for the preparation of nanostructured NbSe_2_ take hours^13,14,16−19^ or even days,^15^ the duration of the present methodology is in the order of minutes (see Table S1 in the Supporting Information). Indeed, after just 2 min of cathodic treatment, bulk NbSe_2_ is transformed into well-exfoliated 2D NbSe_2_ nano-objects (Figure 1) with high structural quality (Figure 2). Furthermore, unlike other methodologies, the present protocol is performed at room temperature and under ambient conditions, not involving high temperatures,^14,16−19^ inert atmosphere,^14,16,17,19^ or complex or scarce reagents or solvents^18^ (see Table S1 in the Supporting Information). Significantly, the final individual exfoliated nano-objects could be directly obtained from the electrochemical treatment without the need of any further substantial energy input (e.g., sonication). Such an outcome was quite different to that found for typical electrochemical exfoliation methods of most layered materials, such as graphite, black phosphorus, and LTMDs, including NbSe_2_ itself. In those cases, the electrolytic treatment usually affords just a bulk expanded material (e.g., worm-like expanded particles or accordion-like expanded crystals), from which individual nanosheets need to be subsequently extracted in the liquid phase via, e.g., sonication or shear forces.^51,52^ In contrast, no expanded particles were generated by the present electrochemical treatment of the bulk NbSe_2_ powder. Indeed, FE-SEM imaging of the gray particles that sedimented at the bottom of the electrolytic cell during the treatment (not shown) showed them to be as compact as those of the starting material (Figure 1b). This suggested that delamination in the present case did not proceed through simultaneous intercalation/expansion of many interlayer galleries in the NbSe_2_ particles, which is the expected exfoliation pattern for common electrochemical methods, but rather via a sequential, layer-by-layer peeling process. The latter would be more similar, although probably not identical, to the exfoliation behavior brought about by direct sonication of the bulk material. However, as noted above, the electrolytic exfoliation rate of NbSe_2_ was much faster than that afforded by direct sonication. These observations pointed to an unusual exfoliation mechanism taking place here, which was thus worth examining.

We hypothesize that the present electrolytic exfoliation of NbSe_2_ proceeded via a singular path akin to the redox mechanism of LTMD exfoliation proposed in recent years.^53,54^ Such a redox exfoliation has been shown to rely on the generation of soluble molecular metal oxide precursors (e.g., orthometalates) on the surface of the LTMD particles, including NbSe_2_, either via naturally occurring oxidation processes under ambient air or by purposeful oxidation with a chemical oxidant. Subsequent reduction of these soluble orthometalates yields the corresponding polyoxometalates. The latter are highly charged polyatomic anions that adsorb on the surface and at the interlayer gaps of the LTMD, triggering its sequential delamination through Coulombic repulsions with just a very small energy input (e.g., stirring) being required. This mechanism provides a priori a consistent conceptual framework to understand the aqueous electrolytic exfoliation of NbSe_2_. Specifically, oxidation-prone NbSe_2_ could certainly provide an abundant source of naturally occurring orthoniobate anions when the starting bulk material has been stored in ambient air, as it was the case here. Indeed, the XPS results shown in Figure 2c provided evidence of the presence of oxidized Nb species in the starting NbSe_2_ powder. It could be argued that, under the intrinsically reductive conditions of the present cathodic treatment, the orthoniobate anions would assemble into polyoxoniobates, and then the latter would drive the sequential exfoliation of the NbSe_2_ particles as discussed above. This delamination process would be assisted by the intercalation of K^+^ ions at NbSe_2_ edges,^27^ affording a wedge effect that would facilitate the adsorption of orthoniobates and polyoxoniobates at interlayer gaps.

It should be noted, however, that the redox mechanism of LTMD exfoliation was not originally proposed in the context of either electrolytic or aqueous systems.^53,54^ Consequently, different aqueous electrochemical processes that were previously not contemplated may have contributed to the currently observed outcome. In fact, we believe that during the aqueous cathodic treatment of NbSe_2_, new oxidized Nb species are continuously generated in situ, facilitating the sequential delamination of the material. In such a scenario, the native Nb oxides already present in the starting bulk powder would not be strictly necessary to make exfoliation possible. To show evidence of this point, the starting NbSe_2_ powder was subjected to reflux in acetonitrile with the aim of removing its native molecular Nb oxide species (this solvent is known to solubilize molecular metal oxide precursors present in LTMDs, including NbSe_2_^53,54^). Then, the refluxed NbSe_2_ material was tested in an aqueous cathodic delamination experiment. If the native molecular Nb oxide species were strictly required for delamination to occur, the yield of exfoliated products obtained with the acetonitrile-treated NbSe_2_ powder should be substantially lower, if not negligible, relative to that attained with its nontreated counterpart. Nonetheless, essentially the same yield was obtained in both cases (i.e., ∼16 wt %), suggesting that in situ-generated Nb oxide species are indeed central to the exfoliation process.

**Figure 3:**
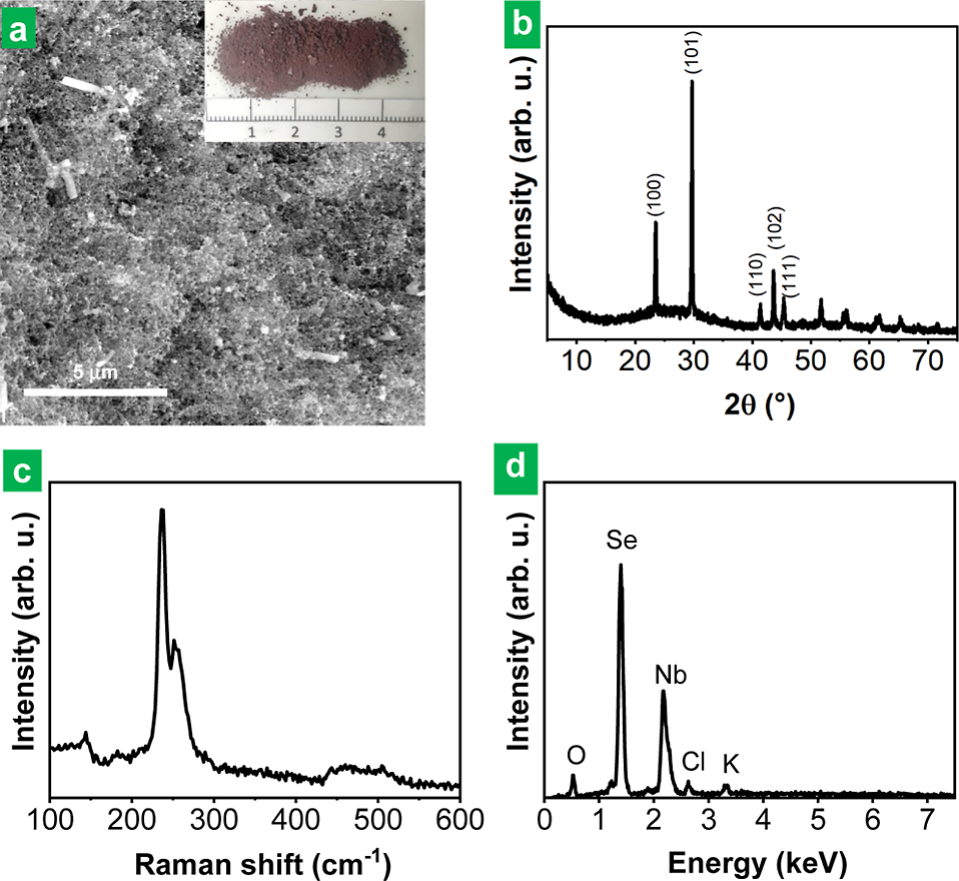
Characterization of the product of the treatment of bulk NbSe_2_ powder with 3 M KOH solution (pH ∼14) at 90 °C for 30 min: (a) FE-SEM image with a digital photograph in the inset, (b) XRD, (c) Raman, and (d) EDX. The XRD patterns obtained show the main peaks characteristic of crystalline t-Se at 2θ values of 23.5, 29.7, 41.4, 43.7, and 45.4° corresponding to the crystal planes (100), (101), (110), (102), and (111), respectively, which have been labeled for clarity.

The generation of Nb oxide species during cathodic delamination most likely resulted from the pH-dependent hydrolytic oxidation of NbSe_2_ and more specifically hydrolysis under basic conditions. We note that due to the high bias voltage applied to the electrolytic cell, water molecules are expected to be reduced at the cathode side, yielding hydrogen molecules and hydroxide anions that raise the local pH of the aqueous electrolyte around the cathode. To demonstrate that a basic medium triggers the hydrolytic oxidation of NbSe_2_, a control experiment was carried out whereby bulk NbSe_2_ powder was stirred in a 3 M KOH solution (pH ∼14) at 90 °C for 30 min. Upon completion of the treatment, a very small amount of gray particles were seen to sediment at the bottom of the beaker and the supernatant solution developed a transparent dark red tone. The material in the supernatant was recovered by flocculation induced by the addition of HCl, washed through several cycles of centrifugation and resuspension in water, and finally dried under vacuum. The resulting material was a dark red powder with a small amount of brilliant gray particles (see the digital photograph in the inset in Figure 3a and FE-SEM image in the same figure) with a weight that amounted to ∼92–94% of that of the starting bulk NbSe_2_ powder. The gray particles that made up the sediment were also collected and washed, but their weight was just a few percent of the initial mass of NbSe_2_ used for the treatment with alkali. XRD confirmed the presence of NbSe_2_ in the gray particles (pattern not shown). In contrast, no sign of the LTMD was noticed in the XRD pattern of the powder coming from the supernatant, where just elemental Se could be detected (Figure 3b). We note that the XRD pattern should come from the gray, crystalline Se allotrope as the red allotrope is amorphous and is thus not detected by XRD. The latter result was supported by Raman spectroscopy (Figure 3c), as only the characteristic peaks of (crystalline gray and amorphous red) elemental Se were seen in this material but not those typical of NbSe_2_. Still, Se was not the sole element present in the dark red powder: energy-dispersive X-ray (EDX) spectroscopy (Figure 3d) revealed that it also contained substantial amounts of Nb (9–24 at %) and O (19–40 at %), together with some residual K and Cl (∼1–2 at %) from the KOH and HCl solutions. Because Se was only found in its elemental form, we infer that O must be combined with Nb, thus forming Nb oxide species.

The above results demonstrate that, under basic conditions, NbSe_2_ undergoes hydrolytic oxidation to give elemental Se and Nb oxide species. Moreover, the alkaline treatment can be made harsh enough to induce an almost complete decomposition of the material. Further hydrolysis experiments carried out at lower KOH concentrations indicated that substantial decomposition of NbSe_2_ only occurred above a threshold pH value of about 10.5. Based on this new understanding and the general mechanism of redox exfoliation of LTMDs, the following course of events can be proposed for the aqueous cathodic delamination of NbSe_2_: (1) under the applied cathodic potential (−10 V), water molecules are reduced at the NbSe_2_ electrode, thus raising the local pH of the electrolyte in a region close to the electrode surface, from an initial value of ∼6 (pH of 0.3 M KNO_3_ solution). (2) The local basic medium induces some degree of hydrolytic oxidation of the NbSe_2_ surfaces that are in contact with the electrolyte, if they were not previously oxidized. Such an oxidation generates molecular Nb oxide species, most likely in the form of polyoxoniobates [e.g., the hexaniobate anion (Nb_6_O_19_)^8–^ referred to as the Lindqvist ion], rather than the monomeric orthoniobate anion, NbO_4_^3–^. This is because the latter is not stable in aqueous medium, whereas polyoxoniobates like the Lindqvist ion are known to readily form under alkaline conditions (pH > 10.5).^55^ (3) An initial cathodic intercalation of K^+^ ions, probably as hydrated entities ([K(H_2_O)_*n*_]^+^),^27^ takes place at layer edges of the NbSe_2_ particles, making the interlayer space at such edges to become exposed to the aqueous electrolyte, which triggers its oxidation. The resulting highly charged polyoxoniobates adsorb on the two opposite surfaces of the interlayer space, generating a strong electrostatic repulsion between them that causes their separation.^53,54^ This event initiates delamination proper of a sheet at its edges. The assisting role of K^+^ ions in the process explains the previously reported influence of KNO_3_ concentration in the delamination yield (Section 2.1). (4) The incipient delamination of the sheet favors the ensuing intercalation of K^+^ ions and the oxidation of the interlayer space at locations increasingly farther away from the edges. As a result, the sheet is progressively delaminated. (5) When delamination is completed, the sheet is released into the aqueous medium and a freshly exposed surface is ready for new exfoliation events. The very nature of the process entails the oxidation of both surfaces of the exfoliated sheets.

The delamination mechanism proposed here implies that the yield of exfoliation should be dependent, in a nonmonotonous way, on the local pH of the electrolyte in the region close to the NbSe_2_ cathode. Although this local pH could not be measured, its actual value during the cathodic treatment was probably not very far from the threshold of 10.5 that was shown above to trigger the hydrolytic oxidation of NbSe_2_ and is also known to allow the solubilization of polyoxoniobates.^55^ If it had been much lower than 10.5, no or little hydrolytic oxidation of NbSe_2_ would have occurred, and so not many polyoxoniobates would have formed to support sheet delamination. As a result, a low exfoliation yield would have been expected. On the other hand, an exceedingly basic local pH would have likely driven a much too aggressive oxidation of the exposed NbSe_2_ sheets, which would have extensively decomposed into elemental Se and Nb oxide species. Consequently, smaller amounts of NbSe_2_ would have been available for delamination, thus leading to lower exfoliation yields. To test this prediction, the local pH around the NbSe_2_ cathode should be controlled. While this is not possible by direct means, some indirect control should be feasible by tuning the pH of the starting electrolyte. To that effect, we conducted additional cathodic delamination experiments using purposefully acidified and basified electrolytes. More specifically, we prepared an acidic (pH ∼1) and a basic (pH ∼11) electrolyte by adding a proper amount of HNO_3_ and KOH, respectively, to the standard 0.3 M KNO_3_ neutral electrolyte, which gave exfoliation yields of ∼6 wt % (acidic) and ∼11 wt % (basic). This result is to be compared with the yield of ∼16 wt % obtained with the standard KNO_3_ electrolyte (pH ∼6) and is therefore consistent with the predictions derived from the proposed delamination mechanism.

The second relevant feature of the exfoliated NbSe_2_ material was the fact that it could be obtained in the form of rolled-up nanosheets (nanorolls). This feature was not exclusive of the present electrolytic delamination process: as noted above, nanorolls could be generated as well just by sonicating the starting NbSe_2_ powder in water, albeit at much lower yields. Moreover, different 1D NbSe_2_ nanostructures, such as nanorods and nanowhiskers, have been previously generated by the processing of bulk NbSe_2_ via, e.g., sonication or ball milling.^56,57^ However, the origin of such a behavior is not clear. Nanoscrolls of several other LTMDs (MoS_2_, MoSe_2_, WS_2_, etc.) have been reported in recent years,^58−61^ but their preparation typically relies on the postprocessing of the LTMD already in 2D form, not in bulk form. Indeed, the attainment of 1D nano-objects directly from the exfoliation of the bulk material has been rarely documented for LTMDs other than NbSe_2_.^62,63^ It is thus reasonable to conclude that the ability of bulk NbSe_2_ to yield 1D nano-objects directly upon exfoliation should be connected to some particular traits of this LTMD. We believe that the relatively strong tendency of NbSe_2_ to oxidize (stronger than that of many other LTMDs) plays a central role. Specifically, the surface oxidation of the exfoliated NbSe_2_ nanosheets is thought to generate stress in their structure in a way similar to the surface stress brought about by the adsorption of organic molecules (ligands) onto colloidal nanoplatelets, including LTMD nanosheets, which drives their folding into curved morphologies.^61,64^ In this scenario, it is reasonable to assume that such a surface stress may be affected by the interaction of the oxide layer with its surrounding medium, and so that the induction of curvature in the nanosheets depends on the solvent where they are dispersed. For the cathodically delaminated material, the required surface oxide species would be generated in situ, as discussed above. On the other hand, for the material obtained by direct sonication, only the native surface oxides would be in principle available to support exfoliation and folding into 1D nanorolls, which would explain its lower yield. However, it should be possible to increase the exfoliation yield by generating oxide species in situ in this case as well, provided that the sonication treatment is carried out under proper alkaline conditions. To this end, we sonicated bulk NbSe_2_ powder at different basic pH values, controlled by the addition of KOH. As could be anticipated, the exfoliation yield increased substantially when working at pH values of ∼10–11, once again confirming the key part played by hydrolytic oxidation in the delamination behavior of this LTMD.

Finally, we demonstrated that the cathodic exfoliation route developed here for NbSe_2_ could be extended to other LTMDs. To this end, metallic LTMDs such as NbS_2_ or VSe_2_ seemed to be better candidates than their semiconducting counterparts (MoS_2_, WS_2_, MoSe_2_, etc.) for two reasons. First, for the present exfoliation strategy to succeed, the LTMD should be highly reactive toward oxidation, particularly alkaline hydrolytic oxidation. By their own nature, metallic LTMDs are generally expected to be more prone to oxidation than semiconducting ones.^65^ Second, the higher electrical conductivity of metallic LTMDs should favor the occurrence of the processes that lead to their exfoliation under electrolytic conditions. Such processes should be much more limited for semiconducting LTMDs as a result of their lower conductivity. Indeed, upon application of a negative voltage (−10 V), bulk NbS_2_ and VSe_2_ resulted in, respectively, gray and brownish-red colloidal dispersions (see Movie S4 and Figure S8a for NbS_2_ and Movie S5 and Figure S8b for VSe_2_ in the Supporting Information) of micrometer- and submicrometer-sized 2D NbS_2_ and VSe_2_ nanosheets (see Figure S8c,d in the Supporting Information, respectively). Thus, we have shown that the present simple and fast exfoliation strategy of low environmental impact is applicable to metallic LTMDs, making metallic 2D LTMDs available for, e. g., EES.

### 2.4 Lithium Storage Performance of the Cathodically Delaminated NbSe_2_ Materials

**Figure 4:**
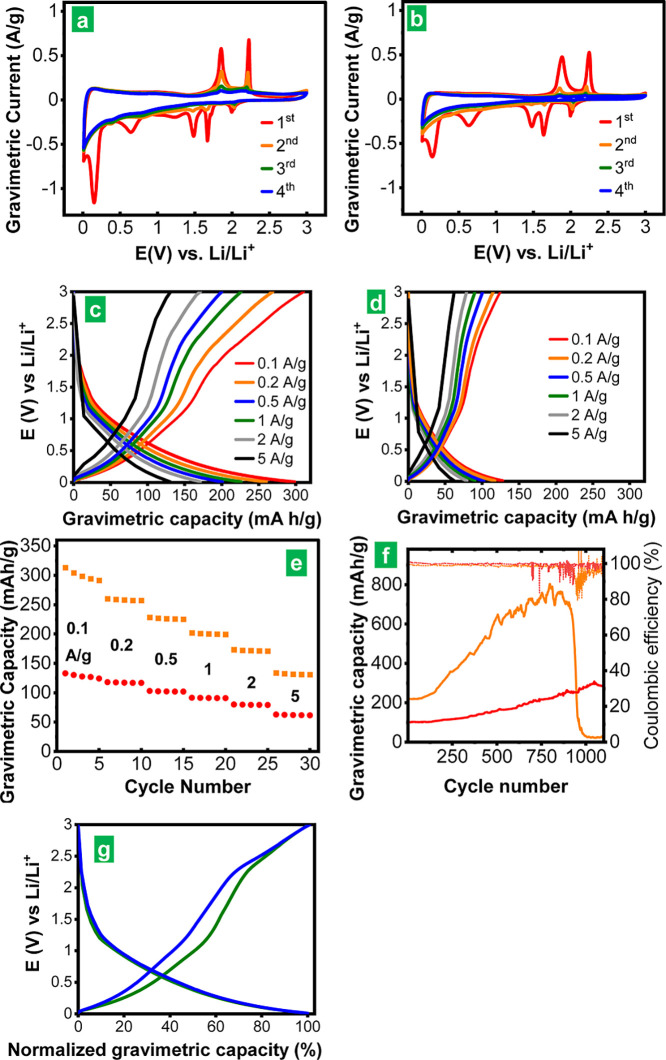
First four CVs for NbSe_2_ nanorolls (a) and unfolded nanosheets (b) at a scan rate of 0.1 mV s^–1^: first (red trace), second (orange), third (green), and fourth (blue). Typical GCD profiles for NbSe_2_ nanorolls (c) and nanosheets (d) at current densities between 0.1 and 5 A g^–1^. (e) Gravimetric capacity values for NbSe_2_ nanorolls (orange squares) and nanosheets (red circles) determined at different current densities from the discharge curves in (c,e), respectively. The gravimetric capacity and current density values are given relative to the total mass of the NbSe_2_-based electrode; the figures would be a factor of ∼1.85 larger if given relative to the mass of active material (i.e., mass of NbSe_2_ only). (f) Cyclability of NbSe_2_ nanorolls (orange trace) and nanosheets (red trace) in terms of capacity (solid trace) and Coulombic efficiency (dotted trace) at a current density of 0.5 A g^–1^. (g) Typical GCD for the early stages of cycling of the NbSe_2_ nanorolls (green traces) and for the electrode with increased capacity after several hundred cycles (blue traces). For ease of comparison, the profiles have been normalized to their full discharge capacity.

The Li storage performance of the two cathodically delaminated NbSe_2_ products, i.e., nanorolls and unfolded nanosheets, was evaluated in a half-cell configuration, with the NbSe_2_ material combined with a conductive additive (a mixture of carbon black and carbon nanotubes) and a binder as the working electrode, Li metal foil as the counter and reference electrode, and 1 M LiPF_6_ solution in an ethylene carbonate/diethylene carbonate solvent mixture (1/1 weight ratio) as the electrolyte (see the Experimental Section in the Supporting Information for details). We note that the two cathodically delaminated NbSe_2_ products were not dispersed as individual objects in any solvent after their preparation and were thus expected to retain their different morphology through their testing as electrodes. Indeed, as will be seen below, their dissimilar electrochemical performance gave experimental indication of the persistence of such morphological difference. Figure 4a,b shows the first four cyclic voltammograms recorded for the rolled-up nanotubes and unfolded nanosheets, respectively, at a scan rate of 0.1 mV s^–1^ in the potential range 0.01–3.00 V vs Li/Li^+^. The CVs of both samples were qualitatively identical and, in agreement with previous reports, they were dominated by a number of redox features characteristic of NbSe_2_ materials, where Li storage is thought to arise from a combination of intercalation and conversion processes.^13−15^ Specifically, in the first cathodic scan (red trace, negative current range), the peaks located at ∼1.64–1.67 and 1.47 V vs Li/Li^+^ have been ascribed to a transition of the NbSe_2_ lattice from the starting H phase to the H́ phase upon Li intercalation (i.e., *x*Li + H-NbSe_2_ → Li_*x*_H́-NbSe_2_), as well as to the conversion of NbSe_2_ into Li_2_Se and metallic Nb (4Li + H-NbSe_2_ → 2Li_2_Se + Nb). Furthermore, the relatively broad cathodic peak at 0.63 V vs Li/Li^+^ is thought to result from the conversion reaction of H́-phase NbSe_2_ [Li_*x*_H́-NbSe_2_ + (4-*x*)Li → 2Li_2_Se + Nb], whereas the sharp, intense peak at ∼0.15 V vs Li/Li^+^ can be traced to the irreversible formation of a solid–electrolyte interphase (SEI) at the NbSe_2_ electrode. In the ensuing anodic scan (red trace, positive current range), the relatively featureless, low-current ∼0.05–1.50 V vs Li/Li^+^ region can be attributed to the conversion of Li_2_Se and Nb back to Li-intercalated NbSe_2_ [2Li_2_Se + Nb → Li_*x*_H́-NbSe_2_ + (4-*x*)Li], while the sharp peaks at 1.86–1.88 and 2.23–2.25 V vs Li/Li^+^ have been attributed, respectively, to delithiation of NbSe_2_ [Li_*x*_H́-NbSe_2_ → H́-NbSe_2_ + *x*Li] and to oxidation of Li_2_Se to give lithium polyselenides and/or elemental Se [e.g., *n*Li_2_Se → Li_2_Se_*n*_ + 2(*n*-1)Li, with 4 ≤ *n* ≤ 8 ]. The latter chemical species are then expected to reduce back to Li_2_Se during the following cathodic scan (orange trace), which was indeed reflected in the peak appearing at 2.00–2.02 V vs Li/Li^+^. Significantly, this peak was already noticed in the first cathodic run of both samples, thus supporting the above conclusion that some elemental Se was present in the as-prepared delaminated NbSe_2_ materials. The intensity of the peaks associated to intercalation/conversion processes in NbSe_2_ decreased after the first CV, which suggested that such processes were not totally reversible.

Quantitative comparison of the CVs for the nanorolls and unfolded nanosheets revealed that consistently higher currents were measured with the former material, implying its higher electrochemical activity (the mass loading of both samples was the same, i.e., ∼0.65 mg cm^–2^). Such a result was consistent with the idea that tubular morphologies should be more conducive to facilitating access of the electrolyte to the active material, compared to the case of unfolded nanosheets:^66,67^ the strong tendency of the latter to restack upon processing should be substantially alleviated when the nanosheets are rolled-up into nanorolls. The larger electrode–electrolyte contact area expected for the rolled-up nano-objects was reflected in the much more intense current associated to the generation of the SEI layer (i.e., the cathodic peak at ∼0.15 V vs Li/Li^+^ of the first CV) in this sample relative to that of the unfolded nanosheets, as can be noticed in Figure 4a,b. Due to the irreversible formation of the SEI layer as well as to the partial irreversibility of the intercalation and conversion events in NbSe_2_, the Coulombic efficiency of the first voltammetric cycle was rather low and even more so for the nanorolls (∼58% vs 62% for the unfolded nanosheets), but in both cases, the efficiency tended to approach 100% upon subsequent cycles.

Typical galvanostatic charge–discharge (GCD) profiles recorded at different current densities between 0.1 and 5 A g^–1^ after the first few initial cycles are presented in Figure 4c,d for the nanorolls and unfolded nanosheets, respectively. As expected, larger capacity values were obtained with the rolled-up nano-objects both at low and high current densities. For example, capacities of ∼300 (nanorolls) and ∼130 (unfolded nanosheets) mA h g^–1^ at 0.1 A g^–1^, as well as ∼130 (nanorolls) and ∼60 (unfolded nanosheets) mA h g^–1^ at 5 A g^–1^ were measured. More detailed data of the rate capability of the samples are given in Figure 4e. In both cases, about ∼45% of the capacity could be retained when the current density was increased from 0.1 to 5 A g^–1^. We note that these gravimetric capacity and current density figures are given relative to the total mass of the NbSe_2_-based electrode. If given relative to the mass of active material (i.e., mass of NbSe_2_ only), the capacities/current densities would be a factor of ∼1.85 larger. Taking this point into account is important when making comparisons with results from the literature, where the distinction between total mass of the electrode and mass of active material in gravimetric data is not always explicitly stated. When such comparisons were possible on an equal footing, the present cathodically delaminated NbSe_2_ material was seen to exhibit a very good performance. Specifically, the Li storage capacities for the rolled-up nano-objects were competitive with recent excellent results from a few-layer NbSe_2_@graphene heterostructure obtained by a wet ball milling technique.^15^ Relative to the mass of active material, the NbSe_2_@graphene heterostructure yielded capacities of 492 and 416 mA h g^–1^ at 0.1 and 3 A g^–1^, respectively, to be compared with values of 545 and 325 mA h g^–1^ at 0.18 and 3.7 A g^–1^, respectively, measured for the present NbSe_2_ nanoroll sample (see Table S1 in the Supporting Information for a comparison of the electrochemical performance of the materials prepared here with the very few examples reported in the literature for NbSe_2_-based materials applied to Li storage).

The cyclic behavior of the delaminated NbSe_2_ materials was also investigated, and the corresponding results (capacity and Coulombic efficiency values) are shown in Figure 4f for tests carried out at a current density of 0.5 A g^–1^ (relative to the total mass of the electrode). Remarkably, the capacity was seen to increase to a large extent after several hundred charge–discharge cycles, especially in the case of the nanorolls (orange solid trace), e.g., it was a factor of ∼3 times higher (up to 750–800 mA h g^–1^) for this sample upon 700–800 cycles, compared to a factor of ∼2 (up to 200–220 mA h g^–1^) for the unfolded nanosheets (red solid trace). Nonetheless, after about 900 cycles, the capacity of the nanorolls plummeted to values below 50 mA h g^–1^ over the course of several tens of cycles (∼0.1% of capacity decrease per cycle), which was indicative of severe cell failure. By contrast, the capacity of the unfolded nanosheets continued to rise up to ∼320 mA h g^–1^ for some additional 300 cycles, reaching a plateau subsequently and starting to decline slowly (∼0.06% per cycle) after 1600 cycles (see Figure S9). The steady increase in capacity upon repeated cycling would appear to suggest that not all of the active materials in the electrode were initially available for Li storage. This could be due to the barrier effect of the binder and/or conductive additive, the contact of which with particles of the active material would hamper access of the electrolyte to the latter, thus compromising their activity.^15^ The local volume changes associated to recurrent lithiation/delithiation of the active particles would likely promote the opening of gaps between such particles and the binder/conductive additive, which in turn would facilitate a more extensive infiltration of the electrolyte. The fact that no such an improvement in rate capability was observed between nanoroll and nanosheet morphology (see Figure 4e) must be related to the protocol used to prepare the electrodes, i. e., casting a mixture of lyophilized powder with additives onto copper foil (see the Experimental Section in the Supporting Information for details), which must favor restacking of the nanosheets, but to a limited extent. If a method more prone to restacking of the nanosheets, such as, e. g., filtering dispersions of the active material to yield films, had been used, the lower tendency to restack the nanorolls would be expected to lead to a relatively better performance in the rate capability tests as well.

It is worth noting, however, that the maximum capacities measured here relative to the mass of NbSe_2_, i.e., ∼1450 mA h g^–1^ for the nanorolls and ∼600 mA h g^–1^ for the unfolded nanosheets, were much larger than the theoretical capacity calculated for this LTMD in bulk form, i.e., ∼427 mA h g^–1^, which was obtained assuming a full conversion to Li_2_Se and Nb. This suggests that Li storage mechanisms other than those described above must contribute heavily to the measured capacities. Similar to the case of graphene with respect to graphite,^68^ the present large excess capacity likely arises from the formation of Li multilayers on the surface of the NbSe_2_ nano-objects and maybe also on the surface of the carbon particles used as a conductive additive. Such a Li storage process would be expected to occur at very low potentials (e.g., below 0.3 V vs Li/Li^+^). Indeed, a large fraction of the measured capacities was seen to come from that potential range (Figure 4c,d). To further explore this question, Figure 4g plots two GCD profiles from the rolled-up nano-objects, one of them typical of the early stages of cycling (cycle no. 20, green traces) and the other one typical of the electrode with increased capacity after several hundred cycles (cycle no. 700, blue traces). An equivalent plot for the unfolded nanosheets is shown in Figure S10. To facilitate comparisons, the profiles have been normalized to their respective capacity, that is, with the full discharge capacity equaling to 100%. If the large excess capacity built up steadily over several hundred cycles was only due to the progressive formation of Li multilayers, we would expect the corresponding discharge profile to be considerably skewed toward very low potentials compared to that obtained during the initial cycles. Nevertheless, as noticed from Figures 4g and S10, that was not the case. Rather, the two discharge profiles were very similar to each other in the two samples. This result implied that the active material in the electrode became increasingly available as a whole upon cycling, i.e., not just the outer surface of the particles for the formation of Li multilayers but also simultaneously the interior of the NbSe_2_ nano-objects for intercalation/conversion reactions.

On the other hand, as also noticed in Figures 4g and S10, the normalized charge profiles skewed toward higher potentials upon prolonged cycling, particularly in the case of the nanorolls, indicative of an increased polarization of the cell. Although the origin of this effect is currently unclear, we believe it may be related to Ohmic processes (Ohmic polarization).^69^ As the gaps between active NbSe_2_ particles and binder/conductive additive progressively open, the quality of the electrical contacts between the electrode components can be expected to decrease, which would be possibly further promoted by the formation of additional SEI layers in the new gaps. This would result in an electrode with poorer electrical conductivity and, consequently, with increased polarization of Ohmic nature. The increasingly poor electrical contacts within the electrode could also be responsible for the capacity decay noticed in the cells. Specifically, the larger surface area associated to the NbSe_2_ nanoscrolls compared to the unfolded nanosheets, while attractive for promoting higher Li storage capacities, would also be conducive to a more extensive opening of gaps. In turn, this would lead to a more drastic deterioration of the electrical contacts within the electrode and thus to a faster capacity decay in the former sample, as is actually seen in Figures 4f and S9. The abrupt morphological changes during charge/discharge would also lead to sudden changes in the electrical contacts, yielding a zigzagging curve, as seen in the latter figures. A more in-depth investigation of the failure mechanisms of this electrode material will be provided in future work, with the aim of proposing strategies that improve its cycle life performance.

## 3 Conclusions

We have demonstrated a simple and expeditious method for the preparation of 2D NbSe_2_ predominantly in the form of either rolled-up nanosheets (nanorolls) or unfolded nanosheets. This method relies on the direct cathodic exfoliation of bulk NbSe_2_ powder in an aqueous solution of a readily available salt, which makes it especially attractive from a practical standpoint. The fact that the exfoliated nano-objects could be directly obtained by the present electrolytic treatment in a very short time (1–2 min) with substantial yields (∼16 wt %) sets this strategy apart from most other top–down methods previously used for the production of 2D NbSe_2_, such as direct liquid-phase exfoliation via sonication (longer processing times, lower yields) and typical electrochemical exfoliation techniques (use of organic electrolytes, post-treatment required to complete delamination). An inquiry into the cathodic exfoliation mechanism suggested that delamination is driven by a partial hydrolytic oxidation of the material in the locally alkaline environment around the NbSe_2_ cathode. This reaction is expected to generate molecular niobium oxide species (e.g., highly charged polyoxoniobates) that prompt the cleavage of thin NbSe_2_ layers from their bulk parent solid, in a process that resembles the redox exfoliation mechanism of LTMDs. As expected from the particulars of the exfoliation mechanism, the methodology could be extended to other metallic LTMDs, such as NbS_2_ and VSe_2_. As an active material for electrochemical lithium storage, the cathodically delaminated products were seen to exhibit very high capacity values, particularly the material with dominant nanoroll morphology. This can be ascribed to a higher accessibility of the lithium storage sites afforded by such a morphology (larger electrode–electrolyte contact area) compared to the material with unfolded nanosheet morphology, where restacking issues probably constitute a barrier in that respect. Overall, the present results make metallic 2D LTMDs available by a simple and fast preparation strategy of low environmental impact, which is expected to expedite their uses in EES and beyond.

## Supporting Information Available

Demonstration of electrochemical delamination of NbSe_2_ bulk material pressed on flexible graphite for the preparation of NbSe_2_ nanosheets (MP4)
Demonstration of the change undergone by flexible graphite alone under the same conditions (MP4)
Demonstration of electrochemical delamination of a pellet of NbSe_2_ bulk material (MP4)
Demonstration of electrochemical delamination of NbS_2_ into NbS_2_ nanosheets (MP4)
Demonstration of electrochemical delamination of VSe_2_ into VSe_2_ nanosheets (MP4)
Experimental section; evidence on the reversibility of the folding of the NbSe_2_ nanosheets into nanoroll morphology through solvent exchange; further physicochemical characterization of cathodically delaminated NbSe_2_; cathodical delamination of other LTMDs (NbS_2_ and VSe_2_); comparison of the electrochemical performance for Li storage of the cathodically delaminated NbSe_2_ with other NbSe_2_-based materials; and additional information of the cyclability of the cathodically delaminated NbSe_2_ materials for lithium storage (PDF)
