# Supplementary material for: NbSe2 Nanosheets/Nanorolls Obtained via Fast and Direct Aqueous Electrochemical Exfoliation for High-Capacity Lithium Storage
Source: ACS Appl Energy Mater. 2023 Jun 16;6(13):7180–93. doi: 10.1021/acsaem.3c00893 (PMC10337822; doi:10.1021/acsaem.3c00893)
Supplement: Supplementary file 6 — ae3c00893_si_006.pdf [file ae3c00893_si_006.pdf]

Supporting Information.

$NbSe_2$ nanosheets/nanorolls obtained via fast and direct aqueous electrochemical exfoliation for high capacity lithium storage

Daniel F. Carrasco[a], Sergio García-Dalí[a,b], Silvia Villar-Rodil[a,]*, José M. Munuera[a], Encarnación Raymundo-Piñero[b], Juan I. Paredes[a,]*

[a]*Instituto de Ciencia y Tecnología del Carbono, INCAR-CSIC, Francisco Pintado Fe 26, 33011 Oviedo, Spain*

[b]*CNRS, CEMHTI UPR3079, Univ. Orléans, 1D avenue de la Recherche Scientifique, 45071, Orléans, France*

* Corresponding author: silvia@incar.csic.es (S. Villar-Rodil),

* Corresponding author: paredes@incar.csic.es (J. I. Paredes)

S1. Experimental section

S1.1. Materials and reagents

Bulk $NbSe_2$ in powder form was acquired from American Elements. Graphite foil (Papyex I980, thickness ~0.5 mm, mass density ~1.1 g cm^{-3}) was obtained from Mersen. Platinum foil (dimensions: 25×25×0.025 mm^3), lithium foil, copper foil, potassium nitrate (KNO_3), nitric acid (HNO_3, 65-67 wt% solution), potassium hydroxide (KOH), potassium chloride (KCl), hydrochloric acid (HCl, 37 wt% solution), N-methyl-2-pyrrolidone (NMP) and isopropanol were purchased from Sigma-Aldrich. 1 M lithium hexafluorophosphate solution in ethylene carbonate/dimethyl carbonate (1/1 weight ratio) (LP30) was procured from Solvionic whereas carbon nanotubes (CNTs) and carbon black (Super C65) were bought from Arkema and Timcal, respectively. Polyvinylidene difluoride (PVDF, Solef) and glass fiber paper (thickness 670 µm) were acquired from Solvay and Whatman®, respectively. Milli-Q deionized water (Millipore Sigma, resistivity: 18.2 MΩ·cm) was used throughout the experiments.

S1.2. Cathodic exfoliation experiments

The electrolytic delamination of $NbSe_2$ was carried out in a two-electrode set-up under aqueous cathodic conditions, using a platinum foil piece as the counter electrode (anode). In a typical experiment, 100 mg of bulk $NbSe_2$ powder were compacted onto a circular piece of graphite foil (10 mm in diameter) by means of a hydraulic press (5 tons applied for 1 min). The resulting $NbSe_2$/graphite foil electrode and the platinum foil piece were immersed in an aqueous 0.3 M KNO_3 solution (25 mL) at a distance of ~2 cm from each other and connected to a DC power supply (E3633A apparatus, from Keysight Technologies) via crocodile clips. Only about one half of the $NbSe_2$/graphite foil piece was actually immersed in the aqueous electrolyte, the emerged half being held with the crocodile clip. Almost immediately upon application of a negative voltage (-10 V) to the $NbSe_2$/graphite foil electrode, a reddish-brown substance was seen to release from it and to get dispersed in the electrolyte. At the same time, gray particles also detached from the cathode and sedimented at the bottom of the electrolytic cell (see Movie S1 in The Supporting Information). After one minute of cathodic treatment, the bias voltage was turned off and the previously emerged half of the $NbSe_2$/graphite foil piece was immersed

in the electrolyte to treat the corresponding fraction of $NbSe_2$ material (again, a bias voltage of -10 V applied for one minute). Finally, the electrolytic solution containing the reddish-brown dispersion was collected (the sedimented gray particles were discarded) and processed to recover this material for subsequent use. To this end, the dispersion was first sedimented either by allowing it to rest undisturbed overnight or by centrifuging it at 100 *g* for 10 min. Centrifugation was conducted with the electrolytic dispersion in glass vials that in turn were inserted into 50 mL centrifuge tubes. This was done to avoid direct contact of the dispersion with the polypropylene material of the centrifuge tubes, as sedimentation of the reddish-brown product caused it to strongly adhere to the latter, which prevented its recovery. Then, the sedimented material was re-suspended in pure water by a brief treatment with a vortex mixer. Following three consecutive sedimentation/re-suspension cycles, the reddish-brown product was subjected to a final sedimentation step, and the sediment was finally collected and dried under a vacuum. For the subsequent studies, this dried product could be readily dispersed in water and isopropanol via a brief treatment (1–2 min) with a vortex mixer or a bath sonicator.

S1.3. Characterization techniques

The materials were characterized by X-ray diffraction (XRD), field emission scanning electron microscopy (FE-SEM), scanning transmission electron microscopy (STEM), atomic force microscopy (AFM), energy-dispersive X-ray (EDX) spectroscopy, Raman spectroscopy and X-ray photoelectron spectroscopy (XPS). XRD patterns were recorded with a D5000 diffractometer (Siemens), using Cu K_{α} radiation, a step size of 0.015º and a step time of 1 s. FE-SEM, STEM and EDX spectroscopy measurements were accomplished with a Quanta FEG apparatus (FEI Company) working at a bias voltage of 20–25 kV. Specimens for FE-SEM and EDX spectroscopy were prepared by mounting the sample (dry powder) onto the sample-holder by means of double-sided carbon adhesive tape, while those for STEM were procured by drop-casting a dispersion of the sample in water or isopropanol (10–20 μL) onto a copper grid covered with a thin continuous carbon film (200 square mesh, MicrotoNano), and allowing it to dry under ambient conditions. AFM images were obtained with a Nanoscope IIIa Multimode microscope (Veeco Instruments) working in the tapping mode of operation. Rectangular silicon cantilevers with a nominal spring constant of 40 N m^{-1} and resonance frequency

of 250-300 kHz were employed. To image exfoliated $NbSe_2$ nano-objects by AFM, the delaminated material was dispersed in isopropanol, drop-cast (10–20 μL) onto a freshly cleaved highly oriented pyrolytic graphite (HOPG) substrate and dried under vacuum at room temperature overnight (drying under ambient conditions led to molecularly thin islands of the alcohol on the HOPG surface, which could be largely removed under vacuum conditions). The recorded AFM images were analyzed with SPIP software (Image Metrology). Raman spectra were acquired with a Renishaw inVia Qontor instrument, working at a laser excitation wavelength of 532 nm (green line). To minimize damage to the sample, the incident laser power was set below 0.5 mW. XPS was carried out on a SPECS system equipped with a Phoibos 100 hemispherical electron energy analyzer. The spectra were recorded at a take-off angle of 90º, working at a pressure below 10^{-7} Pa and using a monochromatic aluminum X-ray source operated at a voltage of 14.00 kV and a power of 175 W. The photoexcited electrons were analyzed in the constant pass energy mode, using a pass energy of 50 eV for survey spectra and 10 eV for high resolution core-level spectra. The surface charging effect was compensated by the use of an electron flood gun operated at 0.4 eV and 0.10 mA. CasaXPS software was used for data processing. Specimens for both XPS and Raman spectroscopy were prepared in the form of continuous, thin films by drop-casting $NbSe_2$ dispersions onto stainless steel discs, which were allowed to dry at room temperature. In the case of the commercial $NbSe_2$ powder, a pellet was prepared by means of a hydraulic press.

S1.4. Electrochemical measurements

The cathodically delaminated $NbSe_2$ materials were tested as electrodes for lithium storage in a coin cell configuration. The working electrodes were prepared by mixing 54 wt% of delaminated $NbSe_2$ as the active material, 16 wt% CNTs and 20 wt% Super C65 as the conductive additives, and 10 wt% PVDF as the binder. A small volume of NMP was added to the latter components and the mixture was transformed into a homogeneous slurry with the aid of a high-shear mixer. The slurry was then cast onto a 24 cm^2 sheet of copper foil and dried at 120 ºC for 3 h. Circular discs 10 mm in diameter were finally cut from the coated copper foil. The total mass loading of the working electrode (i.e., the combined mass of $NbSe_2$, CNTs, Super C65 and PVDF) on the foil was 0.65 ± 0.03 mg cm^{-2}. A piece of lithium foil was used as both the counter and reference electrode. The coin cells were assembled in a glove box under argon atmosphere, with LP30 as the electrolyte and two stacked glass fiber filters as the separator. The measurements were

carried out with a VMP3 potentiostat (BioLogic), recording cyclic voltammograms at different potential scan rates and galvanostatic charge-discharge profiles at different current densities.

S2. Evidence for the reversibility of the folding of the $NbSe_2$ nanosheets into nanoroll morphology through solvent exchange

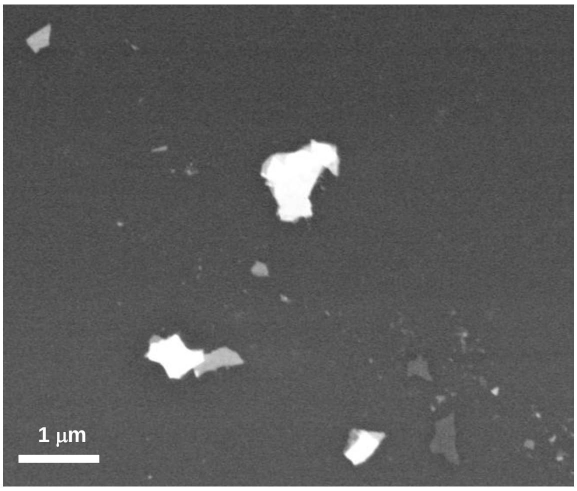

Figure S1. Typical STEM image of the objects coming from a dispersion prepared by suspension of the as-obtained, dried cathodic product first in water followed by transference of the aqueous dispersion to isopropanol (via several cycles of sedimentation by centrifugation and re-suspension in isopropanol).

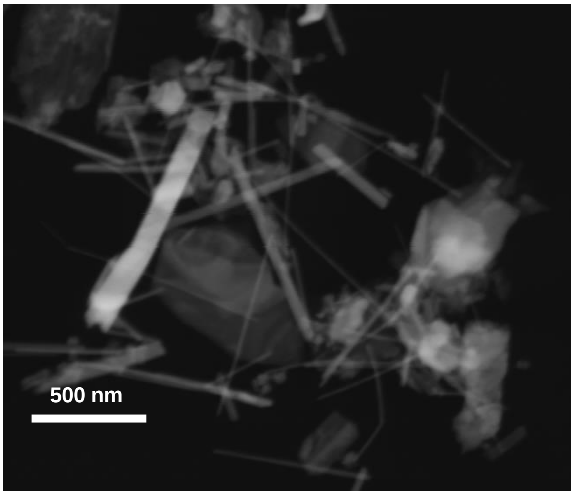

Figure S2. Typical STEM image of the as-obtained, dried cathodic product.

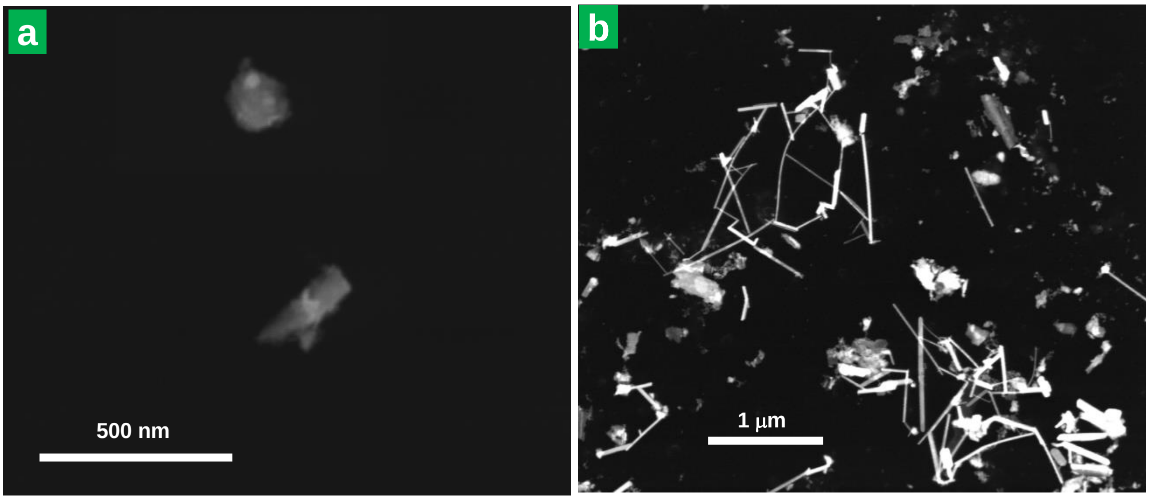

Figure S3. Typical STEM images of the nano-objects obtained by direct sonication of the bulk $NbSe_2$ powder in **(a)** isopropanol and **(b)** water.

S3. Further physicochemical characterization of cathodically delaminated $NbSe_2$

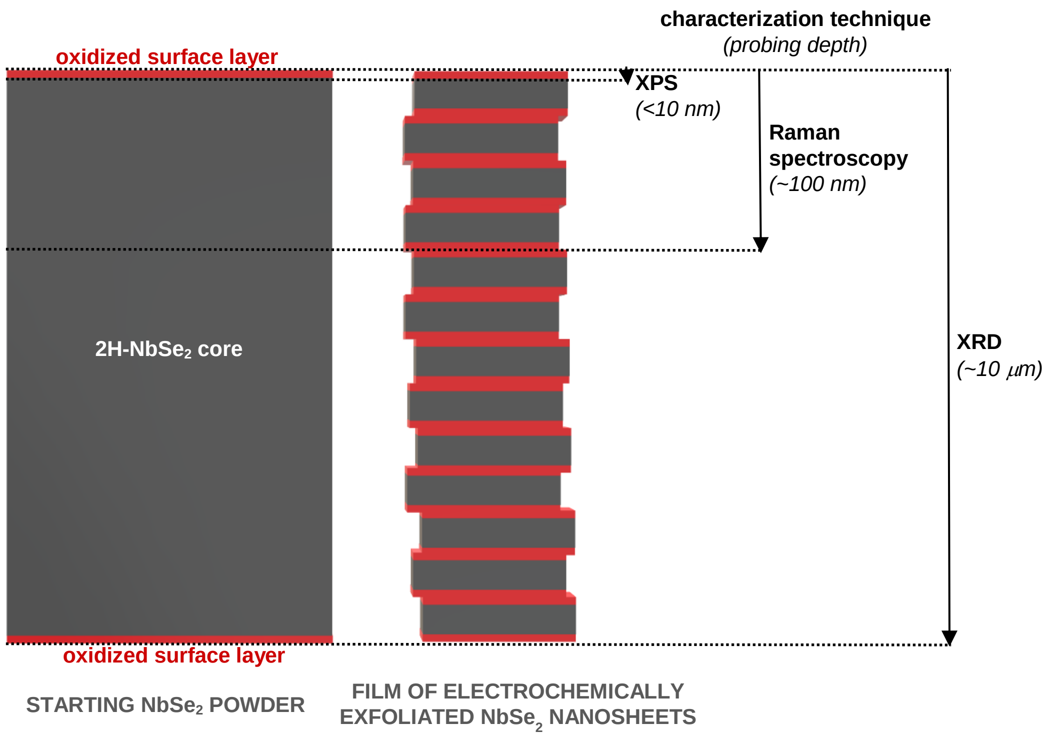

Figure S4. Schematic representation of the structure of the starting $NbSe_2$ powder and that of films of electrochemically exfoliated $NbSe_2$ showing which parts of the structures are probed by different techniques depending on their respective probe depths.

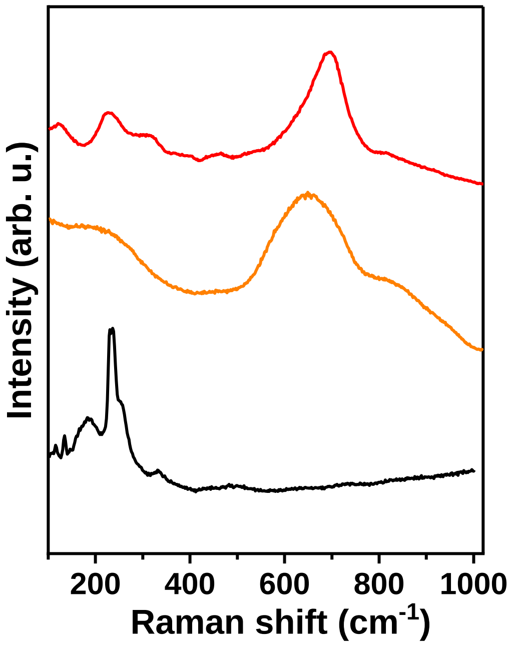

Figure S5. Raman spectra of commercial samples of crystalline (red trace) and amorphous Nb_2O_5 (orange trace), and of cathodically delaminated $NbSe_2$ (black trace).

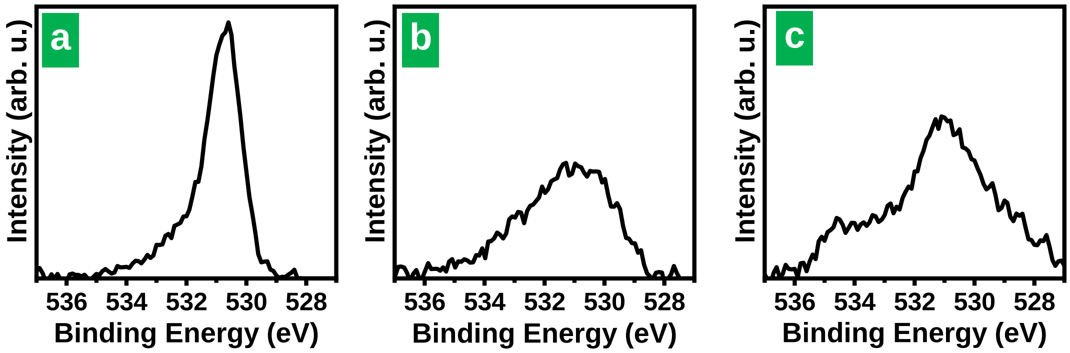

Figure S6. Background-subtracted high-resolution O 1s XPS spectrum of **(a)** the starting bulk $NbSe_2$ powder and the cathodically delaminated product processed in **(b)** water only and **(c)** in isopropanol. The maxima of the O 1s spectra are located ~530.7 eV, which is consistent with the presence of metal oxides.

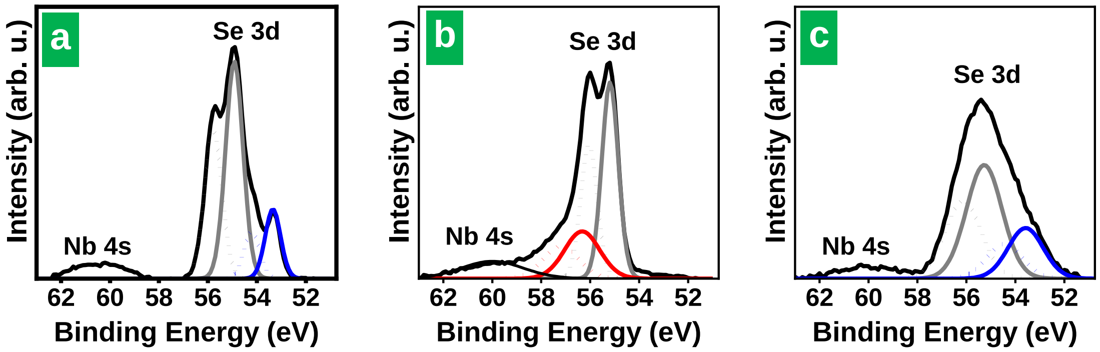

Figure S7. Background-subtracted high-resolution Se 3d XPS spectrum of **(a)** the starting bulk $NbSe_2$ powder and the cathodically delaminated product processed **(b)** in water only and **(c)** in isopropanol. The weak, wide band at binding energy ~60 eV corresponds to Nb 4s core level. Both XPS core level bands have been labelled for clarity. The Se $3d_{3/2}$ and Se $3d_{5/2}$ components are graphed with solid and dotted lines, respectively. Selenium appears in two oxidation states, namely, -2 (blue trace, ~53.3 eV), which is assigned to selenide in $NbSe_2$ [1] and 0 (gray trace, ~55.2 eV), which corresponds to elemental Se [2]. The red component with maximum at 56.3 eV could be also due to elemental

selenium in another less electrically conducting allotropic form, which would become positively charged upon photoemission and consequently shifted to higher binding energy. Indeed, selenium shows different allotropic forms; most of them are non-conducting while the most thermodynamically stable one is electrically conductive [3]. According to the Raman results (see main text) both conducting (crystalline, gray t-Se) and non-conducting (amorphous, red Se) are detected in the surface of the $NbSe_2$ materials.

The fact that some Se in $NbSe_2$ form is detected in the material processed in isopropanol from the Se 3d spectrum (Fig. S7c) but not from the Nb 3d spectrum (Fig. 2e in the main text) can be explained by the difference in the probing depths of the corresponding XPS signals (see Fig. S4). As the kinetic energy of the Se 3d XPS electrons is ~150 eV higher than that of those ejected from Nb 3d core level, the latter band is more surface-specific, and thus more suitable for the detection of surface oxides, while the $NbSe_2$ material underneath is better detected in the Se 3d band.

S4. Cathodic delamination of other LTMDs (NbS_2 and VSe_2)

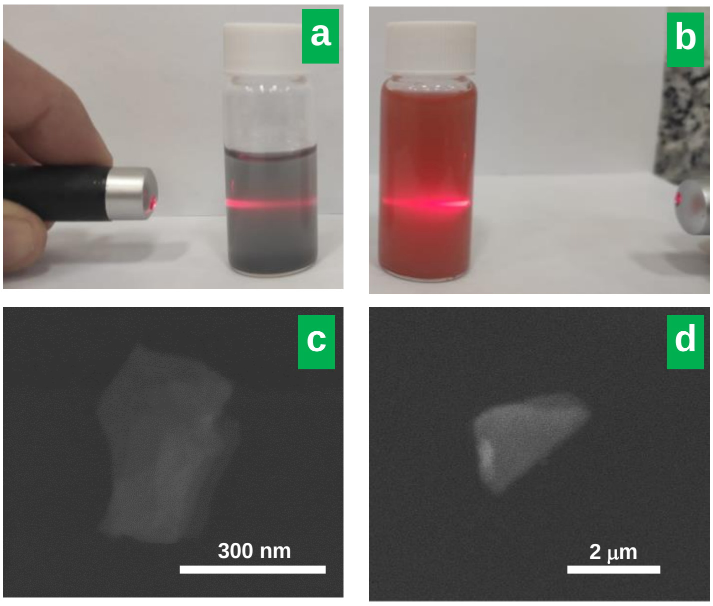

Figure S8. Digital photographs of cathodically delaminated **(a)** NbS_2 and **(b)** VSe_2 in aqueous dispersion showing the Tyndall effect (by which the objects present in colloidal dispersion make the laser beam visible by light scattering). STEM micrographs of **(c)** NbS_2 and **(b)** VSe_2 nanosheets drop-cast from aqueous dispersion on continuous carbon supports.

S5. Comparison of the electrochemical performance for Li storage of the cathodically delaminated $NbSe_2$ with other $NbSe_2$-based materials

There are very few examples in the literature of $NbSe_2$ materials experimentally applied to Li storage. Some other works related to Li storage in $NbSe_2$ in the literature just give proof of the possibility of the intercalation of Li in $NbSe_2$ [4] or deal with theoretical calculations and thus the capacity values provided are not experimental [5–7] and have not been included in the comparison below.

Table S1. A comparison of the performance of different $NbSe_2$-materials for Li storage applications. The gravimetric capacity values are given relative to the active material (i. e., mass of $NbSe_2$ only).

**Material**	**Synthesis method**	**Cyclic performance**	**Rate capability**	**Ref.**
$NbSe_2$ nanorolls	Cathodic exfoliation for 2 min; dispersion in water via a brief treatment (1–2 min) with a vortex mixer	~1450 mAh g^{-1} at 0.9 A g^{-1} upon 700–800 cycles	325 mAh g^{-1} at 3.7 A g^{-1}	This work
$NbSe_2$ nanosheets	Cathodic exfoliation for 2 min; dispersion in isopropanol via a brief treatment (1–2 min) with a vortex mixer	~600 mAh g^{-1} at 0.9 A g^{-1} after 1100 cycles	148 mAh g^{-1} at 3.7 A g^{-1}	This work
Nanosized surface hexagonal $NbSe_2$	Ultrasonic treatment of bulk $NbSe_2$ powder; microwave hydrothermal treatment 180 °C for 12 h	-	313 mA h g^{-1} at 0.1 A g^{-1}	8
$CoSe_2$-decorated $NbSe_2$ nanosheets	Bottom-up, from molecular precursors; cation exchange at 240 °C for 30 min; annealing in Ar atmosphere at 400 °C for 3 h	364.7 mAh g^{-1} at 5 A g^{-1} after 1500 cycles (*)	280 mAh g^{-1} at 10 A g^{-1} (*)	9
Few-layer $NbSe_2$@graphene heterostructure	Wet-ball milling of bulk $NbSe_2$ powder for 40 h	~700 mAh g^{-1} at 1 A g^{-1} after 1000 cycles	416 mAh g^{-1} at 3 A g^{-1}	10

(*) It is not clear whether the reported values are calculated with respect to the active material or to the complete electrode. In case they were given per mass of electrode, the values would have to be multiplied by a factor of 1.43 to express them as relative to the active material.

S6. Additional information of the cyclability of the cathodically delaminated $NbSe_2$ materials for lithium storage

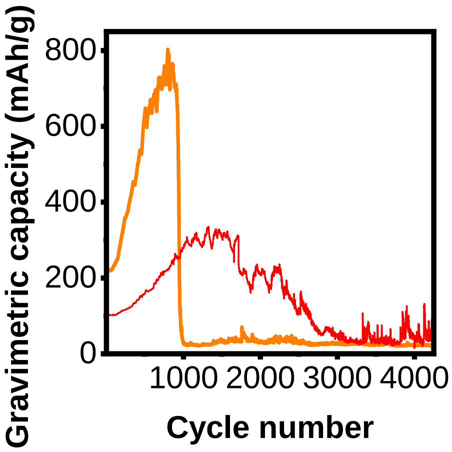

Figure S9. Cyclability of $NbSe_2$ nanorolls (orange trace) and nanosheets (red trace) in terms of capacity at a current density of 0.5 A g^{-1}, including a greater number of cycles (more than 3000) than in the main text. The gravimetric capacity figures are given relative to the total mass of the $NbSe_2$-based electrode; they would be a factor of ~1.85 larger if given relative to the mass of active material (i.e., mass of $NbSe_2$ only).

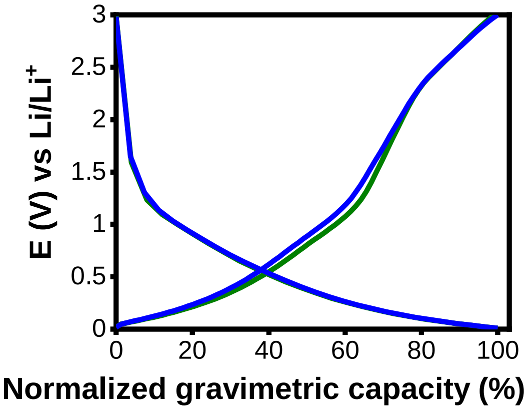

Figure S10. Typical GCD for the early stages of cycling of the $NbSe_2$ unfolded nanosheets (green traces) and for the electrode with increased capacity after several hundred cycles (blue traces). For ease of comparison, the profiles have been normalized to the full discharge capacity.

References

[1] H. Wang, X. Huang, J. Lin, J. Cui, Y. Chen, C. Zhu, F. Liu, Q. Zeng, J. Zhou, P. Yu, X. Wang, H. He, S. H. Tsang, W. Gao, K. Suenaga, F. Ma, C. Yang, L. Lu, T. Yu, E. H. T. Teo, G. Liu, Z. Liu. High-quality monolayer superconductor $NbSe_2$ grown by chemical vapour deposition. Nat Commun 8, 394 (2017).

[2] B. V. Crist. Handbook of Monochromatic XPS Spectra. The Elements & Native Oxides; XPS International LLC: Mountain View, CA, 2004; Vol. 1. p. 245 (Se^0).

[3] N.N. Greenwood, A. Earnshaw, Chemistry of the elements. Ch. 26: Cobalt, rhodium and iridium, Pergamon Press. Oxford, 1984, pp. 886–888.

[] 4 E. Hitz, J. Wan, A. Patel, Y. Xu, L. Meshi, J. Dai, Y. Chen, A. Lu, A. V. Davydov, Li. Hu. Electrochemical Intercalation of Lithium Ions into NbSe2 Nanosheets. ACS Appl. Mater. Interfaces 2016, 8, 18, 11390–11395.

[5] X. Lv, W. Wei, Q. Sun, B. Huang, Y. Dai. A first-principles study of $NbSe_2$ monolayer as anode materials for rechargeable lithium-ion and sodium-ion batteries. J. Phys. D: Appl. Phys. 2017, 50, 23.

[6] H. Liu, Z. Huang, G. Wu, Y. Wu, G. Yuan, C. He, X. Qi, J. Zhong. A novel WS_2/$NbSe_2$ vdW heterostructure as an ultrafast charging and discharging anode material for lithium-ion batteries. J. Mater. Chem. A, 2018,6, 17040-17048.

[7] D. B. Putungan, J.-L. Kuo. Lithium and sodium intercalation in a 2D $NbSe_2$ bilayer-stacked homostructure: comparative study of ionic adsorption and diffusion behavior. Phys. Chem. Chem. Phys., 2021,23, 19811-19818.

[8] C. Peng, H. Lyu, L. Wu, T. Xiong, F. Xiong, Z. Liu, Q. An, L. Mai, Lithium- and magnesium-storage mechanisms of novel hexagonal $NbSe_2$, ACS Appl. Mater. Interfaces 10 (2018) 36988-36995.

[9] J. Zhang, C. Du, J. Zhao, H. Ren, Q. Liang, Y. Zheng, S. Madhavi, X. Wang, J. Zhu, Q. Yan, $CoSe_2$-decorated $NbSe_2$ nanosheets fabricated via cation exchange for Li storage, ACS Appl. Mater. Interfaces 10 (2018) 37773-37778.

[10] H. Nguyen, H. Kim, I.T. Kim, W. Choi, J. Hur, Few-layer $NbSe_2$@graphene heterostructures as anodes for lithium-ion half- and full-cell batteries, Chem. Eng. J. 382 (2020) 122981.
